# Modeling Boundary Vector Cell Firing Given Optic Flow as a Cue

**DOI:** 10.1371/journal.pcbi.1002553

**Published:** 2012-06-28

**Authors:** Florian Raudies, Michael E. Hasselmo

**Affiliations:** 1 Center for Computational Neuroscience and Neural Technology (CompNet), Boston University, Boston, Massachusetts, United States of America; 2 Department of Psychology, Boston University, Boston, Massachusetts, United States of America; New York University, United States of America

## Abstract

Boundary vector cells in entorhinal cortex fire when a rat is in locations at a specific distance from walls of an environment. This firing may originate from memory of the barrier location combined with path integration, or the firing may depend upon the apparent visual input image stream. The modeling work presented here investigates the role of optic flow, the apparent change of patterns of light on the retina, as input for boundary vector cell firing. Analytical spherical flow is used by a template model to segment walls from the ground, to estimate self-motion and the distance and allocentric direction of walls, and to detect drop-offs. Distance estimates of walls in an empty circular or rectangular box have a mean error of less than or equal to two centimeters. Integrating these estimates into a visually driven boundary vector cell model leads to the firing patterns characteristic for boundary vector cells. This suggests that optic flow can influence the firing of boundary vector cells.

## Author Summary

Over the past few decades a variety of cells in hippocampal structures have been analyzed and their function has been identified. Head direction cells indicate the world-centered direction of the animals head like a compass. Place cells fire in locations associated with visual, auditory, or olfactory cues. Grid cells fill open space like a carpet with their mosaic of firing. Boundary vector cells fire, if a boundary that cannot be passed by the animal appears at a certain distance and world-centered direction. All these cells are players in the navigation game; however, their interaction and linkage to sensory systems like vision and memory is not fully understood. Our model analyzes a potential link between the visual system and boundary vector cells. As part of the visual system, we model optic flow that is available to rats. Optic flow is defined as change of lightness patterns on the retina and contains information about self-motion and environment. This optic flow is used in our model to estimate the distance and direction of boundaries. Our model simulations suggest a link between optic flow and the firing of boundary vector cells.

## Introduction

Populations of neurons within the entorhinal cortex and subiculum have firing patterns that depend upon the distance and angle of boundaries in the environment, such as barrier walls. Neurons with this pattern of firing are referred to as boundary vector cells (BVCs) [1]–[3] or border cells [4]. The definition of BVCs includes that of border cells. Border cells specifically fire at a short distance to the wall whereas BVCs fire at a short or long distance to the wall. More general BVCs have a tuning for different wall distances. Boundary vector cells were initially proposed based on observations of changes in the firing location of hippocampal place cells caused by changes in the location of barrier walls surrounding the environment [1], [5]. The initial proposal of BVCs was extended in detailed computational models that explicitly predicted the pattern of firing of BVCs that could, in turn, generate the firing pattern of hippocampal place cells [2], [5], [6]. The predictions of these BVC models have been supported by recent experimental data clearly showing neural firing patterns similar to proposed BVCs in the subiculum [2], [3] and the entorhinal cortex [4], [7].

The firing fields of BVCs at a distance from the walls (Lever et al., 2009) cannot be explained by proximal cues such as those provided by the whisker system. At least three alternative cues could provide the information for distant firing, and these cues are not mutually exclusive. The first possibility is that distance estimates could be retrieved from memory in combination with sensorimotor path integration. This would require the memorization of the entire environment, especially its boundaries. Information about the spatial location of a boundary would be combined with the current spatial position and head direction to estimate distance and direction of that boundary. The current position and head direction of the rat would be estimated from temporally integrated sensorimotor signals. The second possibility is that multiple visual cues on the wall could be used by rats to estimate the normal of the surface and its distance based on the feature's relative size on the projection, requiring knowledge of the absolute size of the feature. However, typical rat experiments lack the presence of distinct visual features, e.g. wallpapers that could be used to estimate the distance of the wall. Therefore, this possibility seems unlikely. A third possibility is the use of optic flow, the varying patterns of light on the retina while the rat is moving. Optic flow could be used for distance and direction estimation of walls based on the following two assumptions: (i) walls are orthogonal to the ground; and (ii) these walls have piecewise smooth surfaces. In this article, we test this “flow-influence” hypothesis by simulating a rat's trajectory in a circular or square box while estimating the distance and direction of walls from optic flow and integrating these estimates into a model of BVC firing. A priori it is unclear if distance and direction estimates extracted from optic flow are accurate enough to support the firing of BVCs. We demonstrate that these estimates are sufficiently accurate, even for drop offs that lack an orthogonal wall.

Further evidence for our flow-influence hypothesis is provided by the rat brain structures processing visual image motion. For instance, neurons in primary visual cortex are sensitive to visual motion [8]. These neurons are tuned for orientation, spatial frequency, and temporal frequency of gratings [9]. Another example is a hierarchy of visual processing identified in rats based on anatomical differences of brain structure. This hierarchy could have similar functions compared to the hierarchy found in primates [10] which is thought to extract properties of optic flow necessary for estimating self-motion [11]–[14]. An alternative pathway that has been explicitly pointed out in the processing of large-field optic flow could go from the retina to the accessory optic system [15] and from there to the hippocampal formation [16]. The latter connection has been described for pigeons. We test this optic flow processing hypothesis and demonstrate that a template model can interpret optic flow patterns and decompose them into variables of self-motion, distance, and direction estimates of walls.

**Figure 1:**
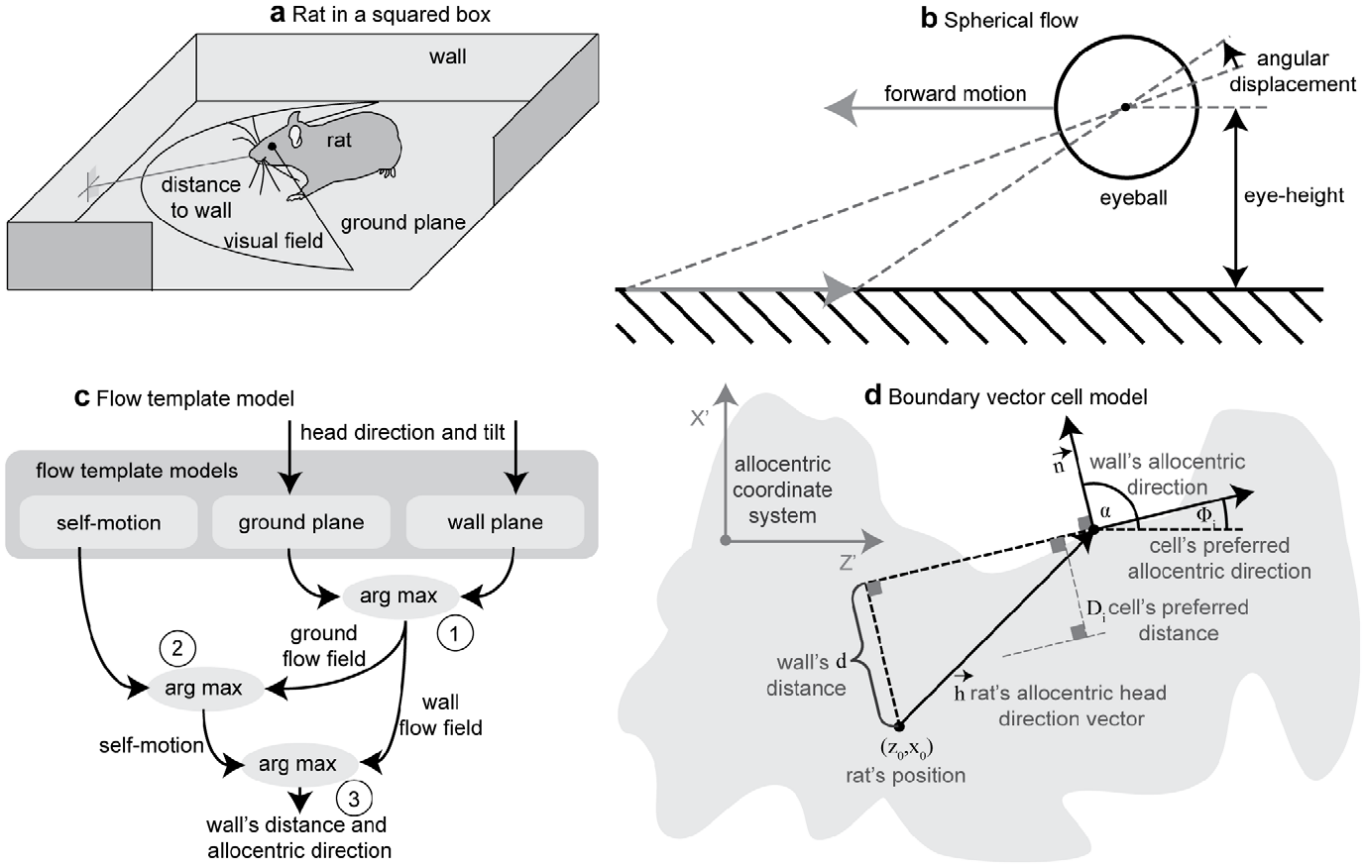
Distance and allocentric direction of walls are encoded in the firing of boundary vector cells. Here we use a model to determine if these cells could be influenced by optic flow. **a**) Shows a rat in a box estimating the distance and allocentric direction of a wall from the sensed patterns of light on its retina. **b**) If the rat moves its eye, e.g. by a forward body motion, these sensed patterns of light shift on the surface of the eyeball. This shift can be described as an angular displacement. **c**) Schematic drawing of the proposed template model. This model sets up flow templates for parameters of self-motion in combination with parameters of planes either describing ground or wall. In a cascade of estimation steps (max-operations) the self-motion and parameters that describe ground and walls are estimated by looking for the best match between flow templates and sensed input flow. **d**) We use a box with an arbitrary outline (gray shading) to display the variables used in the boundary vector cell model. These variables are the cell's preferred allocentric direction *Φ_i_* and preferred distance *D_i_* and the estimated allocentric direction α and estimated distance *d*. All distances are measured with respect to the wall's surface normal.

A sketch of our model is shown in Figure 1. We assume a simulated rat is running in a box, Figure 1a. While the rat is running it samples optic flow patterns from the floor and walls. Sampling is from a wide visual field, as shown in Figure 1a. To model this wide field of view we use a spherical camera model, of which a side-view is shown in Figure 1b. The rat's eyeball is elevated above the ground and is moving in the forward direction, in this example. During this self-motion, sample points of the ground will have an angular displacement in the spherical camera model. The idea of our model is to match all the angular displacements that occur within the visual field by flow templates. These flow templates contain parameters of self-motion, ground, and wall planes, depicted by the three boxes in Figure 1c. Templates of ground and wall are constructed for a specific known head direction and tilt angle of the head, as well as for unknown self-motion parameters. The tilt is the angle between the optical axis and ground. The sensed flow is compared against all of these templates for parameterized self-motion, ground, and wall configurations. In a cascade of steps that detect maximum activity, the model extracts parameters of self-motion and planar surfaces. First, all templates for the ground and wall are compared and a wall-ground segmentation is achieved by selecting the maximum responding template (no. 1 in Figure 1c). Note that the wall and ground template space also contains the parameters of self-motion. Second, the ground flow is used with outputs from the self-motion templates to estimate the self-motion parameters (no. 2 in Figure 1c). Third, the distance and allocentric direction of walls is computed from the wall flow and the parameters of self-motion (no. 3 in Figure 1c). The distance and direction estimates are passed along to the existing BVC model proposed by Burgess [5], [6], [17]. A sketch of the BVC model is given in Figure 1d. The BVC model uses the allocentric direction of a wall together with its distance. Model cells construct a tuning for allocentric direction and distance along the normal direction of the wall. In sum, our modeling work suggests that distance and direction estimates are extracted from optic flow and shows that when these estimates are then fed into the previously developed BVC model this can explain the characteristic firing of BVC cells as measured experimentally [3].

We make several assumptions to focus our modeling effort on the estimation of self-motion, distance and direction from optic flow. First, the visual field, across a full range of angles extending 240° horizontally and 120° vertically, is simulated using a spherical camera model that describes the flow of individual features of the visual scene by temporal changes of the azimuth and the elevation angle of these features (see Figure 1a and 1b). Second, the simulation computes the analytical spherical flow of visual features in a virtual environment instead of estimating flow from an image stream. Third, if the rat is in a rectangular or circular box the walls are orthogonal to the ground. Fourth, we assume that the rat leverages different mechanisms to segment walls from the ground versus detecting drop-offs. Fifth, the model builds upon template cells that are tuned to optic flow that is generated by a combination of self-motion and an environment; and the environment is modeled as smooth surfaces for ground and walls. Tuning for self-motion has been found for neurons in macaque monkeys' area MST [11]–[14]. This finding motivated template models of self-motion estimation in macaque monkeys [18]–[21].

Several aspects distinguish our model from previously published template models. In our model, self-motion is restricted to curvilinear motions: These are translational motions along the optical axis combined with rotations around the vertical axis (yaw-rotations). Our template model uses a spherical camera model that helps to account for effects in large visual fields in contrast to a pinhole camera that is restricted to a 180° visual field. Another difference from existing template models is the introduction of templates that are tuned to the combination of self-motion and smooth surfaces modeling walls or ground. This extended tuning allows not only for the estimation of self-motion but also for the estimation of the distance of these surfaces. We make no assumption with respect to the shape of the box, e.g. it could be square, rectangular, or circular. Note that the introduction of multiple models for ground and wall surfaces also requires the segmentation of flow into these separate surfaces. For instance, a rectangular box consists of a ground plane surrounded by planar walls whereas each individual optic flow sample has to be identified as either originating from ground or wall. Given analytical flow for a spherical camera model, the flow that is induced by linear or rotational motion of a wall can be distinguished from flow that is induced by the same motion of the ground. Thus, segmentation in our model is achieved by deciding whether the wall or ground flow template fits better to the sensed flow vector. Our model provides several extensions to existing template models and is motivated by the need to test whether physiological findings of boundary vector cell firing can be explained by using optic flow as a distal cue.

## Results

We organize our results into four sections. First, we start with the generation of simulated rat trajectories based on the velocity statistics of recorded rat trajectories. These simulated trajectories are used to generate the analytical spherical flow representing the flow that would occur on the rat retina: This flow is then provided as input to our template model. In the second section, we show examples of the wall-ground segmentation and detection of drop-offs with our template model. Third, examples for the estimation of distance and direction of walls are shown, together with the error statistics of distance estimates for an entire simulated rat trajectory. In the fourth section we link our template model to the BVC model and show the resulting firing of model cells compared against data [3].

### Simulated rat trajectories that model rat locomotion

Our aim is to simulate the rat's body movement in an environment similar to the one used in the study of Lever et al. [3]. Therefore, we computed the movement statistics of available rat trajectories in circular [22] and square boxes [23]. Linear velocities are fit by a Rayleigh distribution and rotational velocities by a normal distribution. Values of these fits are reported in Table 1. For these values we generated rat trajectories that matched these velocity distributions. Values of the match are reported again in Table 1.

**Table 1:** Matching statistics of recorded and simulated rat trajectories.

*Reference*	*Files*	*Environment* (a)	*Rayleigh distribution b (cm/sec)*	*Normal distribution μ (°/sec)*	*Normal distribution σ (°/sec)*
Hafting et al. 2005	Hafting_Fig2c_Trial1, Hafting_Fig2c_Trial2, rat_10925	Circular, diameter 180 cm	16.99	−2.48	350.58
Ours	‘CircularCage.mat’	Circular, diameter 79 cm	16.44	0.31	355.35
Sargolini et al. 2006	11084-03020501_t2c1, 11084-03020501_t2c2, 11084-10030502_t1c1, 11084-10030502_t1c2, 11084-10030502_t1c6, 11084-10030502_t3c7, 11084-10030502_t4c1, 11138-11040509_t5c1, 11207-11060502_t6c2, 11207-11060502_t6c3, 11207-11060502_t6c4, 11207-11060502_t6c5, 11207-16060501_t7c1, 11207-21060503_t8c1, 11207-27060501_t1c3, 11343-08120502_t8c2	Square length 50 cm	13.25	0.62	337.93
Ours	‘SquareCage.mat’	Square length 62 cm	13.02	−0.03	330.12
Ours	‘SquareCageWithWall.mat’	Square length 62 cm	12.95	1.89	331.07

(a) Note that the dimensions of the boxes are quite different due to the different sized boxes used in different labs and experiments.

**Figure 2:**
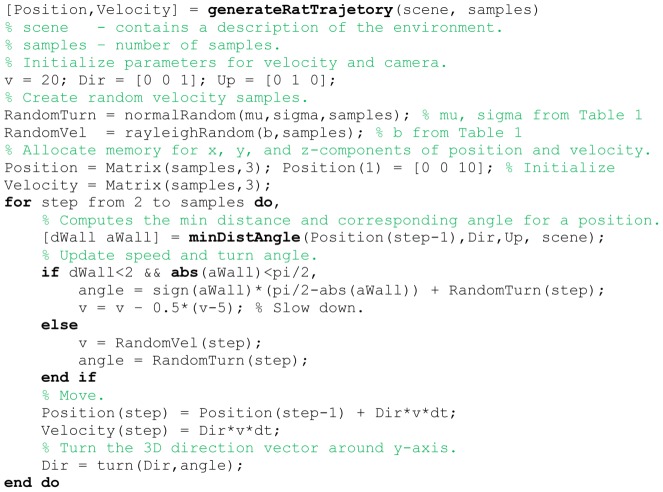
Shows the pseudo-code for the generation of simulated rat trajectories.

For the generation of rat trajectories we combined a deterministic algorithm with a random component. We randomly generate a linear or rotational velocity that follows a Rayleigh or normal distribution, respectively. As the deterministic component we calculate a rotation that turns the rat to continue to walk parallel to the wall. This turn happens only if the rat is closer than 2 cm to the wall and its head direction is smaller than 90° with respect to the normal vector of the wall. Figure 2 contains the pseudo-code for this method.

**Figure 3:**
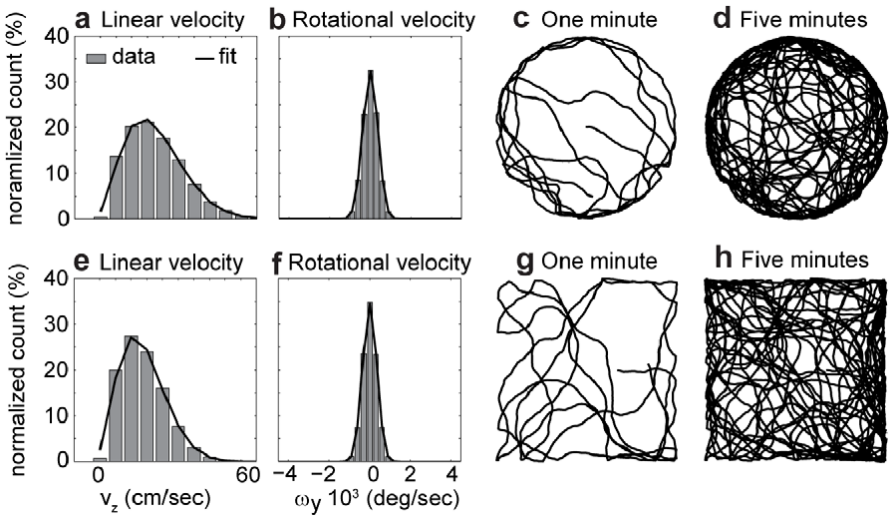
Replication of the velocity statistics of recorded rat trajectories using simulated rat trajectories. **a**) The linear velocities of the rat's body motion are fitted by a Rayleigh distribution. **b**) The yaw-rotational velocities are fitted by a normal distribution. **c**) Shows the first minute of the simulated rat trajectory and **d**) the first five minutes of the same trajectory in a circular box (79 cm diameter). The panels **e**) and **f**) show the fits for linear and rotational velocity for a simulation in a squared box (62 cm×62 cm). The first minute and the first five minutes of the simulated trajectory are shown in **g**) and **h**), respectively.

Figure 3 shows characteristics of our simulated rat trajectories. The Panel 3a shows the Rayleigh distribution of linear velocity (or speed) and Panel 3b shows the normal distribution of rotational velocity for the data for a rat in a circular box. Panels 3c and 3d show fragments of the first minute and of the first five minutes of the simulated trajectory. The second row, Panels 3e–3h, shows the same properties for simulated trajectories in a square box.

### Wall-ground segmentation and drop-off detection

**Figure 4:**
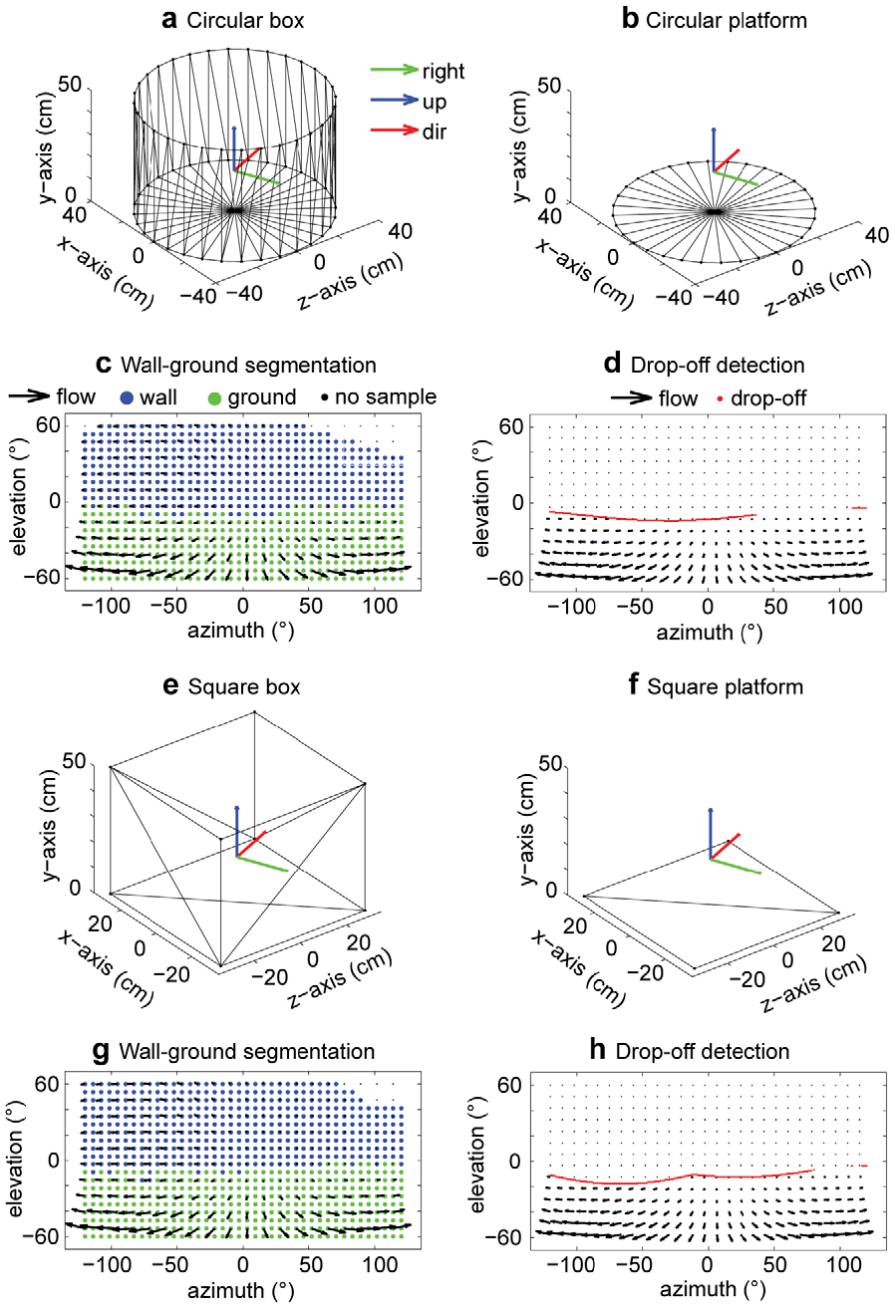
Examples of wall-ground segmentation and drop-off detection by our model. **a**) Depicts a circular box of diameter 79 cm and a 50 cm high wall with the camera at 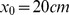, 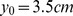, 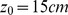, with orientation 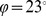, and self-motion 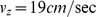 and 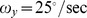. **b**) Shows the same circular box with walls removed to simulate a platform. **c**) Wall-ground segmentation estimated by our model based on the analytical flow shown as black arrows in a). **d**) Drop-off detection based on the flow discontinuities. Note that distant boundary locations are not detected, but these usually do not play a role for behavior. **e**) Shows a square box 62 cm×62 cm with a 50 cm high wall. **f**) Shows the same box as in e) with walls removed. **g**) Estimated wall-ground segmentation for the square box. **h**) Detected drop-off at close distance. In all examples the camera had the same position, orientation, and self-motion as mentioned in a). All boxes are described by a triangular mesh.

Before the distance of walls can be estimated, flow samples of walls have to be segmented from flow samples of the ground. This is accomplished in the first stage of our model, see Figure 1c. Examples of the segmentation are shown in Figure 4c for a circular box and Figure 4g for a square box.

In case of drop-offs our model employs a different mechanism by detecting the flow transition from large to small magnitude. Examples of this detection are shown in Figure 4d for a circular box and Figure 4h for a square box. Note that in these examples the drop-off, indicated by the red dots, is not completely detected. The detection shows gaps where the flow differences are not large enough to be picked up by our mechanism. However, these gaps appear for very distant points of the ground-plane and will not directly influence steering for the rat. In contrast, drop-offs that are close to the rat generate large flow differences that are picked up by our model mechanism and which are potential threats for the rat.

### Estimation of wall distances, irrespective of their surface

**Figure 5:**
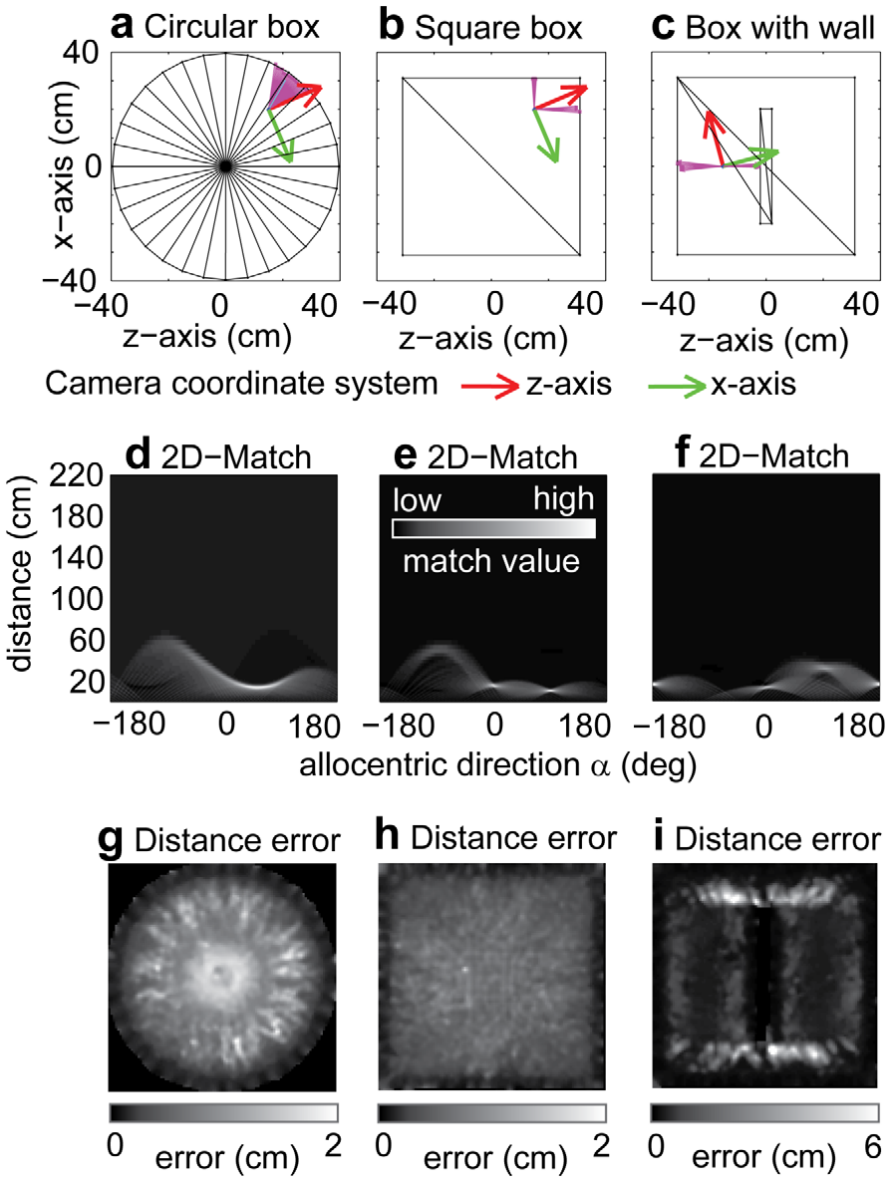
Examples and error statistics for the allocentric direction and distance estimation of walls for different boxes. **a**) Top-view of a circular box with diameter 79 cm and 50 cm high walls. The rat's position is 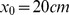, 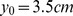, 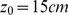 and the camera coordinate system has the orientation 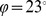 in the xz-plane depicted by the red and green arrows. **b**) Top-view of the same configuration as in a) for a square box with 62 cm×62 cm with 50 cm high walls. **c**) An additional wall has been added inside the square box of b) and the rat's position changed to be 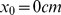, 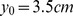, 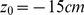 and orientation 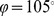. In all cases the camera moved by the linear velocity 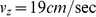 and the rotational yaw-velocity 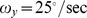. **d**) Match values for distance and allocentric direction of walls in the circular environment (shown in a) provided by our template model. Low match values are encoded by black and high match values by white. **e**) Shows the match values for the square box (shown in b) with same encoding as used in d). Multiple separate regions of high intensity with their peak encode multiple walls as shown in this example. **f**) Match values for the box with interior wall (shown in c). In the last row the mean distance errors over all estimates from 20 min long simulated rat trajectories are shown depending on the position of the rat. **g**) Distance errors for the circular and square box **h**) both range within two centimeters. **i**) For the box with interior wall the mean distance error ranges within six centimeters.

Instead of modeling specific surface types, like curved and planar, we approximate arbitrary surfaces locally by planes. This allows us to use the same model for curved walls of a circular box or planar walls of a square box. Figure 5 shows examples of distance estimates. For instance, in Figure 5a distances are depicted by the magenta colored arrows that closely match up with the boundary of the box. The Panel 5d shows values of the 2D matching function when comparing the sensed flow to flow templates for walls of a certain allocentric direction and distance. The normalized match value is encoded in gray-values whereas black encodes a low match and white encodes a high match. In the example of Figure 5d (circular environment) the maximum is at ≈80° to the right and 20 cm distance. This maximum together with all responses that are within a 70% range of the maximum are displayed in Figure 5a by magenta arrows. Further examples are shown in the 2^nd^ and 3^rd^ column of Figure 5. Note that wall distances are estimated for both curved and planar walls with the same mechanism, as shown in the examples in Figure 5a–c.

Our template model allows for the estimation of distance and direction to multiple walls. In Figure 5b and 5c distances to two walls are estimated. These are represented by their individual matching high intensity regions in Figure 5e and 5f. For instance, for the square box in Figure 5b, high intensity regions appear in Figure 5e at 0° and 90° allocentric direction representing the left and upper wall, respectively. In case of the curved wall in Figure 5a, each segment of the wall is represented by a wall-model. High matching values appear around 80° allocentric direction in Figure 5d. This shows that our model generalizes to non-planar walls.

In addition to these examples of single distance estimates, we evaluated the distance error systematically for each sample point of our simulated rat trajectories of approximately 20 min duration that include 60,000 sample points for the 50 Hz sampling frequency. We compute the mean distance error for each location, computed for all distance estimates made at that location. Note, that this error measure is, mostly, independent of the actual distance to the wall since all positions provide at least two different distances to walls, excluding the center in the circular box or square box. Here, we assume larger distance estimates for the center and smaller ones for areas close to the wall as our model tends to estimate distance to closer walls rather than farther walls. For the circular box, the mean error is largest in its center; see Figure 5g. For the square box the mean distance error is approximately homogenous and smaller, with a value of about one centimeter; see Figure 5h. For the square box with an intrinsic wall the mean distance error has a maximum of 6 cm, occurring at the inner side of the narrow passages at each end of the intrinsic wall; see Figure 5i. Next, we will integrate these distance estimates for allocentric directions into the boundary vector cell model.

### Boundary vector cells might be influenced by optic flow

Optic flow could be one cue to support firing of boundary vector cells (BVCs) that fire for walls being present at a specific distance and allocentric direction. Distant firing distinguishes BVCs from border cells [4]. So far, our template model provides distance and direction estimates of walls, given the allocentric head direction which we assume is available, e.g., from the head direction cell system. The head direction cell system encodes the head direction in an allocentric representation [24]. In our model simulation we assume the head direction and position given by ground-truth values for every sample point. We use the ground-truth head direction to estimate the wall direction in allocentric angular coordinates. Ground-truth positions are used to spatially register the firing of cells in the model. In the corresponding experiment of rats foraging in a box this ground-truth location is given by tracking the rat's position reconstructed from video recording of a light-emitting diode attached to the rat. Ground-truth position values are not provided to our template model of brain mechanisms for the estimation of self-motion or wall distances. When the model produces inconsistent distance estimates, the plotting of these estimates in relationship to ground-truth position appears as noisy plots of firing. Such firing lacks the consistent tuning properties for allocentric direction and distance toward the wall that is characteristic of data on the firing of boundary vector cells [3].

**Figure 6:**
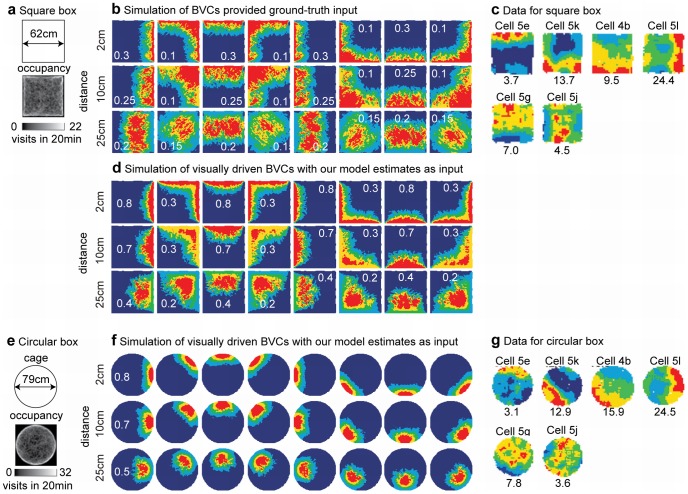
Boundary vector cell (BVC) responses (rate maps) for a square (top) and circular (bottom) box for the model and from data. **a**) Shows the square box and the occupancy that is high at edges for the simulated rat trajectory. **b**) Rate maps for the BVC model using ground-truth distance and direction of walls. For the model we used eight allocentric directions ranging from east, east-north, north, … to south-east combined with the three distance tunings 2 cm, 10 cm, and 25 cm. High firing is encoded as red color and low as blue color. This color encoding is the same for all plots showing firing rate maps. White numbers are the individual scaling parameters for each plot similar to the firing rate scaling used for plotting the experimental data. **c**) Example of recorded BVCs. These firing maps have been redrawn from Lever et al, J. of Neurosci. 29, 2009 from their Figure 3 on page 9774 [3]. The numbers in black denote the firing rate of the cells. **d**) The BVC model receives estimates about allocentric direction and distance from our template model. **e**) Shows the circular box and occupancy of the simulated trajectory. **f**) Shows the rate maps of the BVC model that uses distance and direction estimates of our template model. **g**) Data from recorded BVCs [3].

We compare the data of recordings of BVCs [3] and simulations of the BVC model [5] based on ground-truth input to our visually driven model of BVCs. Figure 6a shows the square box used for this simulation together with the occupancy of the simulated rat in this box. Figure 6b shows the firing of the BVC model when supplied with ground truth input. Figure 6c shows experimental data from recordings of recorded BVCs and Figure 6d shows our visually driven BVC model based on optic flow input. The firing fields of our visually driven BVC model are more restricted in location than the experimental data or the firing of the original BVC model that uses ground-truth input. In the original BVC model only four distance and direction values are used to update the firing of a model cell. For our visually driven BVC model more than four distance and direction estimates are used to update the firing of a model cell, see e.g., the number of magenta arrows in Figure 5a–c. Because there are more estimates incorporated into the visually driven BVC model its firing fields appear more restricted compared to those of the original BVC model. Simulations and data for a circular box are shown in Figure 6e–g. Again, our simulated firing fields appear more localized than the firing of recorded cells. Our model assumes analytically defined flow. However, in case of flow detected from an image sequence, distance estimates could be more erroneous leading to the firing observed in recorded data.

**Figure 7:**
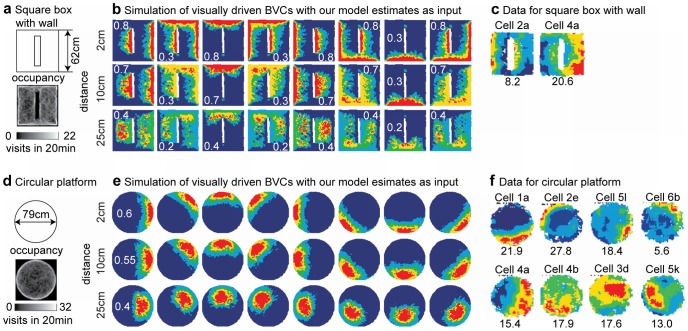
Boundary vector cell (BVC) responses in a box with an additional interior wall and for a platform. **a**) Square box with an additional interior wall and the occupancy of the simulated rat trajectory. **b**) Simulation of the BVC model using estimates from our template model. The tuning of model cells is the same as in Figure 6. **c**) Data of recorded BVC. Note that model and recorded BVCs respond to any wall of a certain distance and allocentric direction and not only, e.g. to the exterior walls of a box. **d**) Circular platform together with the occupancy of the simulated rat trajectory. For reasons of comparison we use the same trajectory as in the simulation with a circular box. **e**) Shows the rate map of model BVC supplied with estimates about distance and direction of walls. **f**) Data from recorded cells. Firing maps of BVC have been redrawn from Lever et al, J. of Neurosci. 29, 2009 from their Figure 3 on page 9774 [3].

Aside from using a square and circular box we provide additional simulations with a wall inserted inside of the square box and in another simulation we removed all walls to model a platform. Figure 7b shows the BVC firing of our simulation and 7c the corresponding experimental data. The important observation from this simulation is that the BVC firing in the model is not tied to a specific wall of allocentric direction and distance but to *any* wall of an allocentric direction and distance. In our simulation, firing appears also next to the inserted wall. In the same way our model cells would adapt to wall changes in the environment as shown in another experiment which involved nesting two boxes, a small one in a bigger one. After some time the smaller box is quickly removed in that experiment. Then firing of BVC shifts its absolute position in the larger box to resemble the same distant tuning that it had in the small box [7]. Our model would produce results consistent with this experiment.

For a platform, the drop-off is detected as a local discontinuity in flow direction and speed. Once the elevation of the drop-off is determined, it is converted into a distance estimate. All distance estimates are fed into the BVC model with their response values. Figure 7e shows the simulation results and 7f the corresponding experimental data. As in previous cases, the BVC in the model is more clearly restricted in location compared to experimental data. This greater restriction in location might differ if the optic flow signal were detected from visual input instead of analytically defined flow that is used in the simulation. In particular, flow detected from visual input would be noisier, and this would influence the accuracy of detection of flow discontinuities.

## Discussion

This paper presents a template model for scene-segmentation and the estimation of geometric properties of the environment, namely the distance and allocentric direction of walls and drop-offs. Distance estimates of our model in empty boxes are accurate within a two-centimeter-range; for a square box with an inserted intrinsic wall the error is higher at locations at the inner edge of the narrow passages created at either end of the intrinsic wall. When these distance and direction estimates are integrated into the boundary vector cell model [2], [5], [6], the typical firing patterns found in experimental data on boundary vector cells can be observed.

Template models for the estimation of ego-motion have been used mainly as a model for self-motion estimation in primates [18]–[21], [25]. All these models used a pinhole camera model. In contrast, here we used a spherical camera model to simulate the large visual field of rats. Existing template models account for general self-motion sometimes restricted by visual fixation, which allows the translational motion to be compensated by a rotation in order to keep a single point stationary in the visual field [19]. No previously published template model provides a link to estimate environmental variables such as distance toward walls. Thus, our model is novel for defining an extended template space and for combining this with voting that allows for the estimation of multiple walls. An advantage of such a voting technique is the more robust estimation and compact description of the surrounding environment in contrast to reconstructing a depth map with variable depths for every single flow vector as suggested by others, e.g. by Perrone & Stone [19].

Most studies on firing properties of hippocampal structures in rats focus on visual cues in general, e.g. a cue card, but not on optic flow as such. For instance, visual cues influence the orientation and firing location of hippocampal place cells [26]–[28]. In neurophysiological recording data on place and head direction cell firing, landmark cues have been shown to dominate over idiothetic cues (e.g. path integration of self-motion information) if the mismatch between cues is smaller than 45°. Above 45° mismatch, the hippocampal representation of place cell firing reorganizes and head direction cell firing is dominated by idiothetic cues [28]. When deprived from vision and audition the majority of place cells (11 out of 15) lose their spatially consistent firing. Instead their firing pattern rotates with the associated arm of a multi-armed maze [29]. Entorhinal lesions had a similar effect to vision deprivation. Sixteen out of 17 place cells lost their spatially consistent firing [30]. Grid cells rotate their firing with visual landmark cues [22]. Combining results from the lesion and sensory deprivation study suggests a role of visual and auditory sensory signals in spatially consistent firing. However, none of the existing studies focused on optic flow as the only cue for spatially consistent firing.

Although visual input provides a rich set of information, other cues might be important for BVC firing, as well. Vestibular information and visual motion influences hippocampal place cell firing [31]. Vestibular inputs are used to find a path back to the home location, especially in the dark [32]. Head direction cells are regulated by the vestibular system [33]. In blind rats place cell firing occurs and in four out of 15 cells the firing is spatially consistent [32]. The consistent firing in four cells provides evidence for the use of idiothetic cues such as path integration in order to maintain a stable representation of the self in the environment model [34; page 466]. Idiothetic cues like path integration and external cues like landmarks interact to regulate place field firing in rats on a running track foraging while cues are brought into mismatch by spatially shifting the goal location [35]. These various examples show that cues other than vision are important to maintain the firing of place cells.

Three alternative technical solutions are possible for the segmentation of walls from ground, and subsequently the estimation of self-motion and environment variables. These are: RANSAC [36], m-functions [37], or the expectation maximization (EM) algorithm [38]. RANSAC could be based on a model for flow of the ground while treating flow samples from walls as outliers assuming that the majority of flow samples originate from the ground. Once the segmentation is achieved all the points identified as outliers can be used to estimate the distance and direction of walls. An integration of Equations (3) and (4) into convex m-functions leads to a non-linear optimization problem. An embedding into the EM algorithm with Gaussian mixture models leads again to a non-linear optimization. Overall, the segmentation of walls from ground is a challenging and computationally expensive task.

For real-life images the quality of flow based segmentation depends largely on the quality of the detected flow and the dissimilarity between flow templates or flow vectors at the drop-off. Since we do not know the quality of detected flow for real-life images we study simulated noise superimposed on the analytically defined flow. Examples with additive Gaussian noise in each component of the flow with a signal-to-noise ratio of approximately 70 dB leads to larger errors in distance and direction estimates (see Figure S1). A major source for this error is insufficient segmentation. Since the segmentation is based on local information, a single flow vector, it is strongly influenced by noise. This could be compensated by adding a neighborhood function into the process of segmentation that assumes neighboring points belong to the same planar model, either wall or ground. Another problem is a close similarity between flow templates if matching a noisy input flow. Therefore, the dissimilarity or “distance” between templates should be maximized in the sense of the proposed matching functions in order to match noisy input flow to the correct flow template. For drop-offs the dissimilarity between flows at the drop-off versus everywhere else in the flow field matters. If discontinuities within the flow due to noise become too large false detections happen. This can be only compensated for with context information, e.g. providing extended curve models for the drop-off in the spherical camera model that could be fitted as an entire curve ranging from −120° to +120° azimuth angle for the parameterization of our spherical camera model. So far, these extensions have not been realized in the current model and are the subject of future work.

Further properties of our model are the logarithm used in the matching function and the model's capability to incorporate tilt angles. Choosing a logarithmic sampling and the logarithm of motion speeds to compare input flow vectors and template flow vectors makes sense for a first-person perspective from an ecological and behavioral point of view. Typically, objects' distances that are far do not have to be represented with a high sampling, e.g. of centimeter-precision, because they are not reachable or are not potential obstacles. A logarithmic sampling of distance values also has an effect on the comparison between flow vectors of different distance. For optic flow generated by translational self-motion the length of flow vectors is inversely proportional to the distance of a sample point in 3D space. By transforming these distances using a logarithm we put more emphasis on short flow vectors that relate to points that are close to the rat. Figure S2 shows a comparison between a matching function that uses the logarithm of the speed and their difference or only the difference of speeds without the logarithm. In both cases the speeds are computed from the input flow vectors and template flow vectors. The matching that includes the logarithm appears clearer over the entire range of depths compared to directly using the difference of speeds. Note that the speed difference that does not involve the logarithm can be adjusted only to accommodate a small depth range with clear tuning. This concept of using a logarithmic sampling and logarithmic scale to compare speeds could be used even more broadly by mechanisms that afford an ecological solution, e.g. if only a limited small number of samples are available.

Another property of our model is the incorporation of non-zero tilt angles. In such configurations the optical axis is not parallel to the ground. The normal vector that describes the wall or ground becomes dependent on the head direction. In our model this head direction is assumed to be given, e.g. by the vestibular cues captured by the head direction cell system, as is the tilt angle (see also Figure 1c). Then our model constructs flow templates for this given tilt and head direction. Simulation results for BVC firing look similar to the ones of Figure 5 and 6 as shown in Figure S3; however, distance errors at large distances are slightly increased. Note that for the positive 30° tilt more flow samples originate from the ground which could give an explanation for the increase in the measured distance error. This is especially the case at large distances to the wall. These two properties of our model, the logarithm of speeds used in the matching function and the non-zero tilt angle that introduces a dependency on head direction, are important for the distance estimation and generalization to other configurations of varying tilt.

Our current model has several limitations. So far, our model responds only to visible walls and drop-offs; however, place cells that may be driven by BVCs also respond in the presence of transparent walls [26]. Furthermore, this model does not work in the dark since our model relies on optic flow, the changes of light patterns on the retina. Another limitation of our model is the restriction of self-motion to curvilinear path motion. Such motions exclude pitch and roll rotations and translational motions that are not parallel to the ground. These limitations could be relaxed by modeling more degrees of freedom in the template model. However, such an extension will increase the number of flow templates. Furthermore, it remains unclear if detecting the separation between ground and wall is still possible for such an extended model in the way it is possible for curvilinear motion. Another restriction of our model is the assumption about analytical, noise-free flow. In reality, flow has to be estimated from light changes and flow estimates would contain errors. To address these limitations future work could include other systems, such as distance estimates from binocular vision, a landmark system along with a triangulation strategy, sensorimotor integration and memory to operate in the dark, or the suggested neighborhood function to improve segmentation given noisy, detected flow.

Information about self-motion and environment structure that is extracted by our model from optic flow could be useful for other cell types as well. Grid cells can be generated by temporally integrated linear and rotational velocities that are estimated from optic flow [39]. Such integration allows for a reasonable estimate of the rat's position in the environment for a short duration, less than a minute with a temporal sampling frequency of 50 Hz. Optic flow can provide the information about short paths and, thus, has the potential to contribute to the place cell firing, a firing tied to specific allocentric spatial location in the environment. The integration of rotational yaw velocities can provide a head-direction signal, again for the time frame of about a minute. Furthermore, there may be an indirect effect as boundary vector cells might influence the firing of grid cells and place cells. Recent studies suggest that BVCs may function as an independent system from grid cells, as inactivation of the medial septum with muscimol causes a loss of grid cell spatial periodicity with sparing of some cells that look like BVCs, and sparing of the spatial firing response of place cells [40], [41]. Thus, optic flow may provide input to cell populations in entorhinal cortex, subiculum, and hippocampus.

Following our “flow-influence” hypothesis our model would predict cells with sensitivity to large flow fields. However, instead of in hippocampal or related areas, we assume this sensitivity to exist in sensory related areas, such as the primary visual area or higher level visual cortical areas or the accessory optic system. In primates these sensitivities have been found in area MT and MSTd [11]–[14]. The spatially integrative behavior of cells can be tested by using motion stimuli of different retinal size while measuring the response from our hypothetical motion cells. Then there should be an effect on firing rate coupled to retinal stimulus size. Furthermore, the “flow-influence” hypothesis for BVC is supported by our modeling work. An experiment testing this hypothesis would record BVCs from subiculum while the animal is passively watching the visual input of a simulated trajectory. To only provide optic flow cues the displayed stimulus would consist of a random dot texture as used in virtual environment setups for humans and should be compared with performance when viewing a display that consists mainly of object outlines that provide visual cues other than optic flow [42]. This passive watching setup should be compared to the freely moving animal while recording from the same BVC – this might be difficult to achieve but testing of virtual environments with stationary animals has been done [43]. Our modeling work would predict that BVC firing will be observed during the passive watching setup; however, we assume it would be nosier, due to the lack of other cues and the prediction that multimodal sensory cues are usually integrated by BVCs during normal behavior.

## Model

We divide the explanation of our template model into the following steps: First, we define the spherical image flow model for curvilinear self-motion. In the second step, flow templates are defined for a ground-plane and planar walls. Third, tuning or matching functions for the comparison between input flow and template flow are defined. Then we summarize all computational steps in an algorithm. Fourth, to interpret and visualize the representation of our template model we define a read-out method. Fifth, a description for the integration of estimated distance and direction values into the boundary vector cell model is given. Parameters of the spherical camera model, template model, and boundary vector cell model are summarized in Table 2.

**Table 2:** Parameters of the models and their values used in the simulations.

*Description of parameter*	*Identifier and value*
**Spherical camera model**	
Horizontal field of view	240°
Vertical field of view	120°
Eye-height above ground	3.5 cm
Horizontal resolution	80 samples or 400 samples^a^
Vertical resolution	40 samples or 200 samples^a^
Minimum distance to samples	0 cm
Maximum distance to samples	1000 cm
**Template model**	
Standard deviation for ground samples	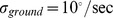
Standard deviation for wall samples	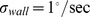
Interval for linear velocities	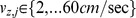
Samples for linear velocities	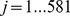
Standard deviation for rotational velocity	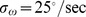
Interval for rotational velocities	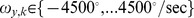
Samples for rotational velocities	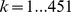
Standard deviation for speed tuning used for walls	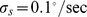
Standard deviation for direction tuning used for walls	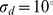
Offset for direction tuning of walls	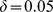
Interval for walls' angles	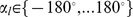
Samples for walls' angles	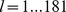
Interval for walls' distances^b^	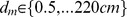
Samples for walls' distances	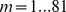
**Boundary vector cell model**	
Standard deviation of distance tuning	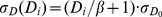
Parameters of distance-dependent tuning	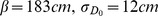
Samples for distances	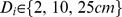
Standard deviation of angular tuning	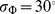
Allocentric direction samples	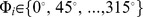

a We use the latter increased sampling for the simulation with drop-offs only to increase the sampling of distances that is coupled to the number of elevations.

b The interval is sampled at a logarithmic scale.

### Spherical image flow model

The spherical image flow model for instantaneous motion through a rigid stationary environment is [44], [45]:
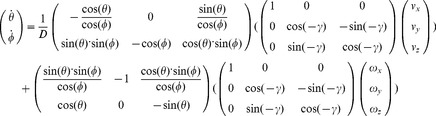
(1)where 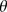 denotes the azimuth angle and 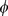 the elevation angle. Azimuth is measured from the z-axis pointing forward along the optical axis in the xz-plane. Elevation is measured from z′-axis in the yz′-plane where z′ denotes the z-axis that is rotated by the azimuth angle. This definition uses a left-handed coordinate system. The 3D linear velocity 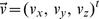 and the 3D rotational velocity 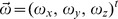 cause temporal changes for azimuth 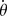 and elevation 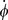 assuming a differential motion model that neglects higher order temporal differences, like accelerations [46], [47]. The super-index ‘t’ denotes the vector-transpose. The distance 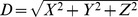 is the length toward a 3D sample point 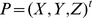 in Cartesian coordinates.

**Figure 8:**
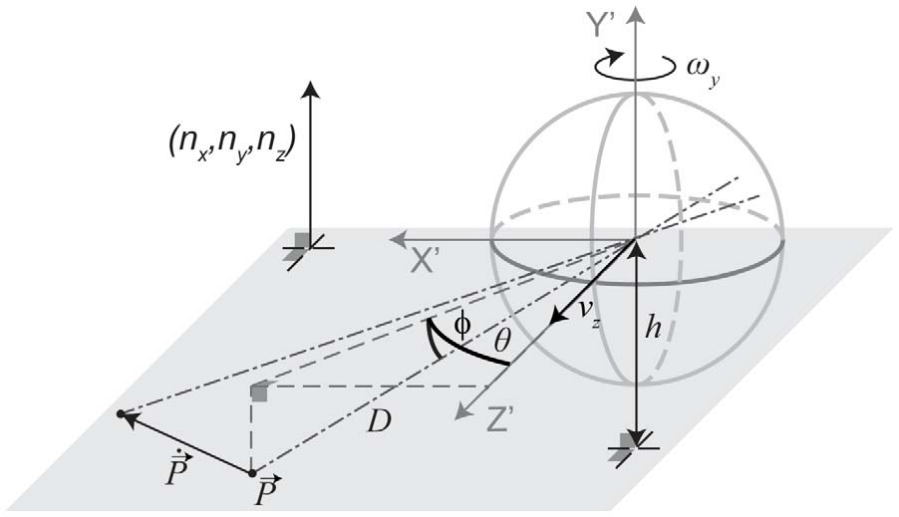
Drawing of the spherical camera model and an analytical flow vector 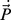 that arises if the entire model is moving by the linear velocity 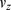 and rotating around the y-axis by 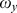. For this paper we use the left-handed-coordinate system with the x-axis 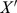 pointing to the right, the y-axis 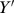 pointing upward, and the z-axis 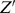 pointing forward. The location 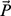 is described by the angles 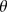 and 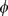 together with its distance 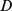 from the origin. Our spherical model describes the flow vector 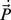 by its angular, temporal differentials 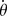 and 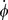 - not depicted in the drawing for clarity.

In the simulations we assume that the rat is moving tangent to the recorded trajectory in the 2D plane. This assumption reduces the six degrees of freedom of the model to two degrees of freedom: The linear velocity 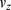 along the optical axis (z-axis) and the rotational velocity 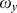 around the y-axis (yaw-rotation). Thus, Equation 1 reduces to a model of visual image motion for curvilinear self-motion:
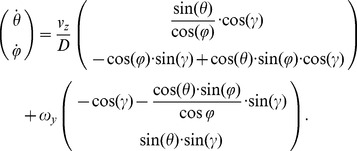
(2)In this Equation 2 the distance variable 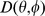 is very general and can be different for every image location defined by the azimuth angle 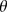 and elevation angle 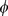. To constrain this variable further, we define a model of a ground plane and planar walls. Figure 8 visualizes this simplified spherical flow model with only two degrees of freedom together with the definition of the camera system.

### Flow template for a ground-plane

In Hessian normal form a plane is described by its unit normal vector 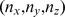 and distance 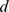. This distance is measured along the normal. Plugging the plane definition into the projection function for the spherical camera model defined in azimuth angle and elevation angle results in the definition of the 3D point distance:
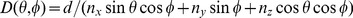
(3)For a ground-plane with 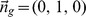 for zero-tilt *γ* = 0 and distance 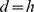 as eye-height above the ground this ground-plane model simplifies to 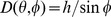. For a tilt angle *γ*≠0 the normal vector is given by 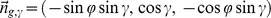 which depends now also on the allocentric camera or head direction *ϕ*. This normal vector 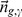 can be computed, e.g., by using Rodrigues rotation equation and rotating the normal vector 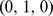 around the axis 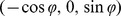.

### Flow templates for planar walls

The depth function for planar walls assumes a wall to be defined by the normal 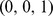 that is rotated according to the allocentric direction *ϕ* of the rat's head which results in 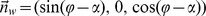 with the angle *α* being the allocentric direction of the wall. For a tilt angle *γ*≠0 the wall's normal vector is described by
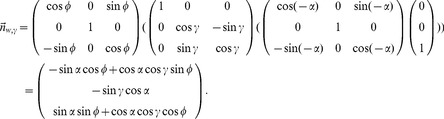
(4)For this definition the order of rotations is crucial: First, we rotate for the wall's direction *α*, second for the tilt angle *γ*, and third by the allocentric direction of the rat's head *ϕ*.

The distance function from Equation (3) with the corresponding normal vectors is plugged into Equation (2) to define the template flows for curvilinear self-motions defined by 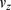 and 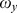. This results in the constrained flow equation:
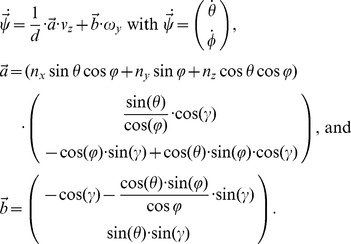
(5)For the normal vector 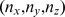 the corresponding model for a ground-plane or wall-plane is plugged in. Tuning functions are employed to compare single flow vectors of the template flows against its corresponding vectors of the input flow. These tuning functions are described next.

### Optimizations and tuning functions of the template model

Input flow is defined as 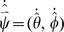 and is compared against the template flow for walls 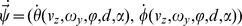 or 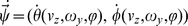, the template flow for the ground-plane where the latter two parameters of distance and angle are dropped. Our first goal is to segment the flows into samples from ground or wall. To derive a flow constraint that is independent of the rotational velocity *ω_y_* but depends on the distances *D*, we multiply the Equation (5) by the vector 
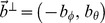
. This provides the following tuning functions for segmentation. First, the tuning function for potential sample points of the ground-plane is:
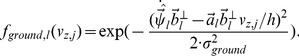
(6)


Second, the tuning function for potential sample points of walls is defined by:
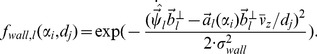
(7)In this tuning function we use the mean velocity 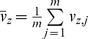 that is computed over all *m* velocity samples. For the wall-ground segmentation we use the following decisions to define the set of wall samples 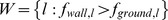 and the set of ground samples 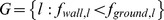.

Then we continue with the ground samples to estimate the linear velocity of the rat by using the tuning function:
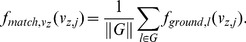
(8)This function in Equation (8) defines matches between the input flow and the template flows for the linear velocity samples 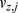. Matches are computed over all samples that have been identified to originate from the ground. This provides the overall similarity between the input flow and a template flow.

Next, we compute the rotational velocity from ground samples. For this computation we use the following tuning function which computes the Euclidean distance between input flow and template flow:
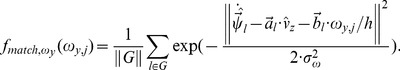
(9)In this Equation (9), 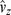 is the estimated linear velocity from Equation (8), e.g. 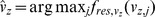.

In the last step we estimate wall distances for a given allocentric direction and use the already estimated linear and rotational velocity from Equation (8) and (9). For this distance and direction estimation we use the tuning function as defined by Perrone [18]. Note, this function has not been used for any of the previous problems due to optimizing for the rotational velocity in Equations (6)–(8) which uses a constraint that is independent of rotational velocities and in Equation (9) because the rotational velocity is independent of the depth and, thus, a more elaborate log-distance tuning as suggested by Perrone [18] for the length of flow vectors is not necessary. But now, since we estimate the distance of walls this distance tuning is crucial. Perrone's tuning model starts with a transformation of flow vectors from Cartesian into polar coordinates, whereas the radius is associated with the speed of an image location. In this polar representation the matching function is defined as:
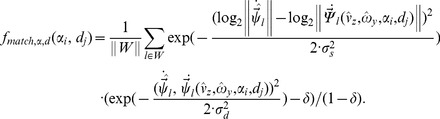
(10)The Equation (10) combines the log-speed tuning, the first factor, with the direction tuning, the second factor, by multiplication. The angular difference in Equation (10) is denoted by 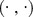. This assumes that the two tunings for motion speed and direction are independent [18]. The already estimated linear velocity 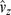 and rotational velocity 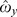 are used to define self-motion specific flow templates in Equation (10).

**Figure 9:**
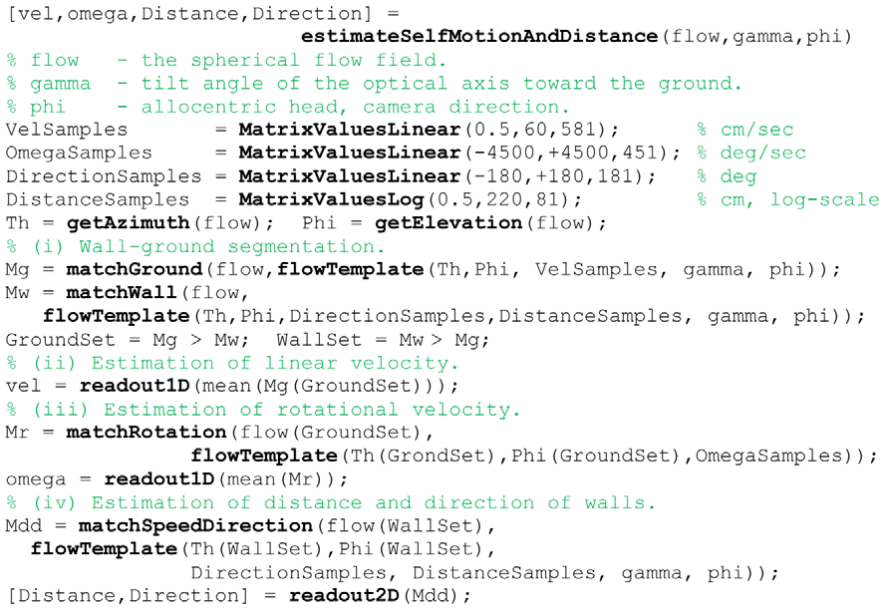
Shows the pseudo-code for wall-ground segmentation, and estimation of self-motion, distance, and direction of walls. The constructor “MatrixValueLinear(min,max,num)” provides a linear equidistant sampling between “min” and “max” of “num” samples. In contrast, the constructor “MatrixValuesLog(min,max,num)” implements a logarithmic sampling between “min” and “max” with “num” samples. The function “matchGround” implements Equation (6) and “matchWall” implements Equation (7). Both functions are used to compute a wall-ground segmentation. The linear velocity is estimated using Equation (8). Further, “matchRotation” implements Equation (9) and “matchSpeedDirection” implements Equation (10). The readout functions “readout1D” and “readout2D” are defined in Code-box 3.

Our extended model can be summarized into the following four steps. First, we compute a wall-ground segmentation by using the tuning functions from Equation (6) and (7). The segmentation is determined by whether a flow vector fits better to a ground template vector from Equation (6) or a wall template vector from Equation (7) while sampling all possible linear velocities in Equation (6) and all possible allocentric directions and possible distances for a wall in Equation (7). Therefore, the segmentation is computed without knowing the parameters of self-motion. But once the segmentation into wall-ground is known we use ground samples to estimate linear and rotational velocity in step two and three, respectively. For estimating linear velocity we use the tuning function from Equation (8) and for rotational velocity the tuning function from Equation (9). In the fourth step, we estimate distance and allocentric direction of walls using the known segmentation, linear, and rotational velocity. A pseudo-code of the algorithm is provided in the Figure 9.

So far, we have not described how velocities, distances, and directions are estimated given the activity from evaluating the residual functions in Equation (8), (9), and (10). Such a description follows.

### Read-out of matching functions

The matching functions in Equation (8), (9), and (10) depend on different stimulus parameters. For instance, the function of Equation (8) depends on linear velocity samples, whereas the function of Equation (10) depends on distance and direction of walls. Our read-out distinguishes between 1D and 2D functions. For a 1D function our read-out method uses a weighted sum with two percent of all argument values that are centered on the maximum. For the 2D match function of Equation (10) we use a different method. Our read-out mechanism selects all matches with their value being within the 70% range with respect to the maximum match. These match values together with their respective arguments, in the above example the linear velocities, are passed along to the distance error calculation or BVC model. The calculation of distance errors takes the direction arguments and computes the ground-truth distance for this direction. Then this ground-truth distance is subtracted from the estimate. The absolute value is computed for this difference to define the distance error.

**Figure 10:**
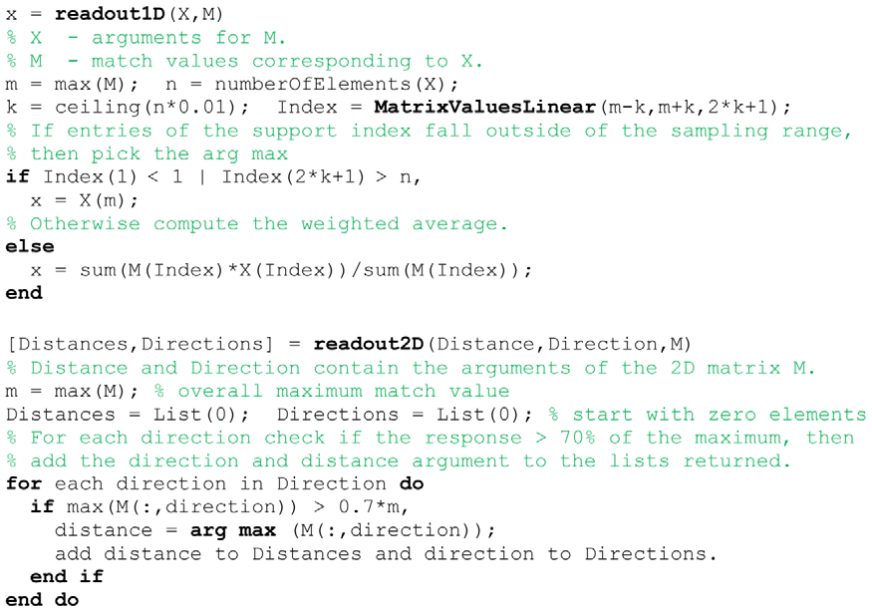
Shows pseudo-code for the interpretation of match values.

If the boundary vector cell model is the next stage, arguments about distance and max read-out directions are passed together with their activation. Distance and direction are integrated into the existing BVC model and we weigh each BVC activity by the match value provided by our template model. This is described in more detail next. A pseudo-code of the 1D and 2D readout method is given in Figure 10.

### Integration of wall's direction and distance estimates into the boundary vector cell model

The boundary vector cell (BVC) model was described in detail elsewhere [5], [6], [17]. Here, we only repeat the main model equation to show how our estimated variables are integrated. The distance 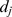 with its allocentric direction 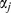 of a wall leads to the activation:
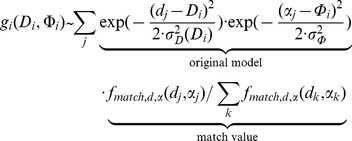
(11)We assume the *i*-th BVC is tuned to the distance 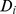 and the allocentric direction 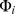. Normalized match values of the template model are included in the firing of a BVC. These match values are the third factor of the product in Equation (11). The indices *k* and *j* range over all read out activations from our model that are above 70% of the maximum activity. Parameter values for this Equation (11) and all other equations are reported in Table 2.

### Detection of drop-offs

**Figure 11:**
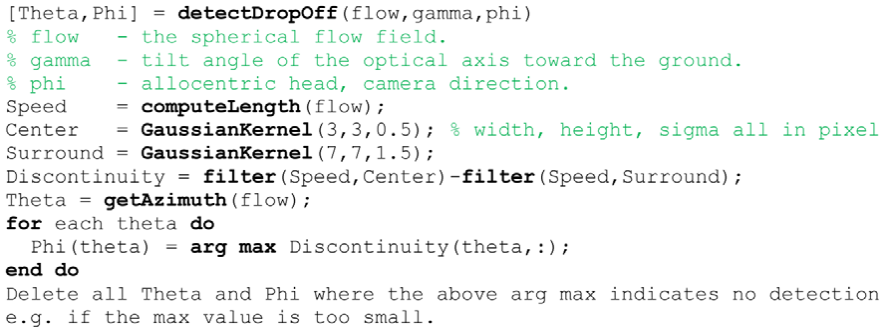
Shows pseudo-code for the method that detects drop-offs.

Drop-offs are detected with a center-surround filter applied to the speeds of the flow field. This detection method assumes that the azimuth and elevation angles are arranged on a regular sample grid that is associated with pixels. In our model example we use 400 horizontal samples and 200 vertical samples. To detect the flow discontinuity at the drop-off we apply a center-surround filter kernel to the length of the flow vectors and detect its maximum response. Formally, this is expressed by:
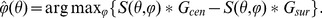
(12)In some cases of small flow discontinuities at the transition from flow at the horizon to the flow of the background this detection does not provide valid values indicated by a small maximum response, see also the pseudo-code in Figure 11.

The detected elevation angle of the drop-off is then converted into a distance estimate assuming the eye-height *h*, tilt angle *γ*, and head direction *ϕ* are known:
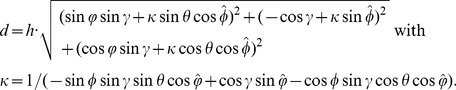
(13)


## Supporting Information

Figure S1Examples of wall-ground segmentations, distance, and direction estimation of walls for analytical flow superimposed with additive, independent Gaussian noise. Flow with noise is defined as 
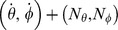
 with *N_θ_* and *N_ϕ_* drawn from a normal distribution with zero mean and *σ_n_* as standard deviation. This type of noise strongly influences the quality of the segmentation and, thus, influences the other estimations based on this segmentation. In both examples the rat is positioned at *x_0_* = 20 cm, *y_0_* = 3.5 cm, *z_0_* = 15 cm, has the head direction *ϕ* = 23°, and the self-motion *v_z_* = 19 cm/sec and *ω_y_* = 25°/sec. **a**) Square box with the coordinate system. The distance estimates are indicted by magenta arrows. **b**) Spherical flow field with noise (*σ_n_* = 5°/sec) and wall-ground segmentation. **c**) 2D match values as calculated from samples that are indicated as originating from a wall, these are the blue dots in b). Low match values are encoded by low intensity and high match values by a high intensity, see also the inset with the color code. **d–g**) 1D match values for the linear and rotational self-motion and the direction and distance of walls. The latter two curves are computed as the maximum response from the 2D match value function of c), whereas the maximum is computed for the dimension not shown. The estimated self-motion is *v_z,est_* = 19 cm/sec and *ω_y,est_* = 24°/sec and the mean distance error is 1.15 cm with a standard deviation of 0.52 cm. **h**) Circular box with coordinate system and distance estimates of the wall depicted by the magenta arrows. **i**) Spherical flow with noise (*σ_n_* = 15°/sec) and wall-ground segmentation for this circular box. **j**) 2D match values. **k–n**) Match value functions for velocities of the camera and direction and distance of walls. The estimated self-motion is *v_z,est_* = 19 cm/sec and *ω_y,est_* = 25°/sec and the mean distance error is 3.03 cm with a standard deviation of 1.29 cm. The distance error in the circular cage is higher due to the assumption about a planar approximation for each segment of the curved wall.(TIF)

Figure S2 Taking the logarithm of the speeds of input and template flow vectors as the argument for a Gaussian function provides a “sharper” tuning compared to taking only the difference of speeds without the logarithm. **a**) Shows the spherical flow field and the detected wall-ground segmentation. **b**) 2D match values for the matching using the log-speed difference shows three clearly distinctive high intensity regions. These are better visible in the break-down into 1D curves **c**) for the wall's direction taking the maximum of all distances and **d**) the wall's distance taking the maximum of all directions. The mean error in distance estimates is 2.76 cm with a standard deviation of 1.81 cm. **e**) 2D match values for the matching using the speed difference without applying the logarithm. Compared to b) the matching occurs fuzzy, also visible in the break-down in the matching for **f**) the walls' directions and **g**) the walls' distances. In this case the mean distance error is 3.74 cm with a standard deviation of 3.85 cm and, thus, higher than in b). This example uses a rectangular box 250 cm×280 cm with 50 cm high walls and the rat's position is *x_0_* = 0 cm, *y_0_* = 3.5 cm, *z_0_* = 10 cm with the head direction *ϕ* = 15°.
(TIF)

Figure S3 A tilt angle of 30°, that is the angle between the optical axes compared to the horizontal parallel to the ground, does no change firing fields qualitatively; however, the error at larger distances is larger compared to a zero tilt angle. **a**) Simulated BVC firing for a square box with distance and direction estimates for walls provided from our template model. **b**) Mean distance error in the two-centimeter range. **c**) Simulated BVC firing for the same square box as in a) but with an additional intrinsic wall. Firing appears at any wall of a specific distance and allocentric direction the cell is tuned for. **d**) Mean distance error in the range of zero to six centimeters. **e**) Simulated BVC firing for a circular box. **f**) Mean distance error in the two-centimeter range.
(TIF)
