# Supplementary figures and images for: Modeling Boundary Vector Cell Firing Given Optic Flow as a Cue
Source: PLoS Comput Biol. 2012 Jun 28;8(6):e1002553. doi: 10.1371/journal.pcbi.1002553 (PMC3386186; doi:10.1371/journal.pcbi.1002553)

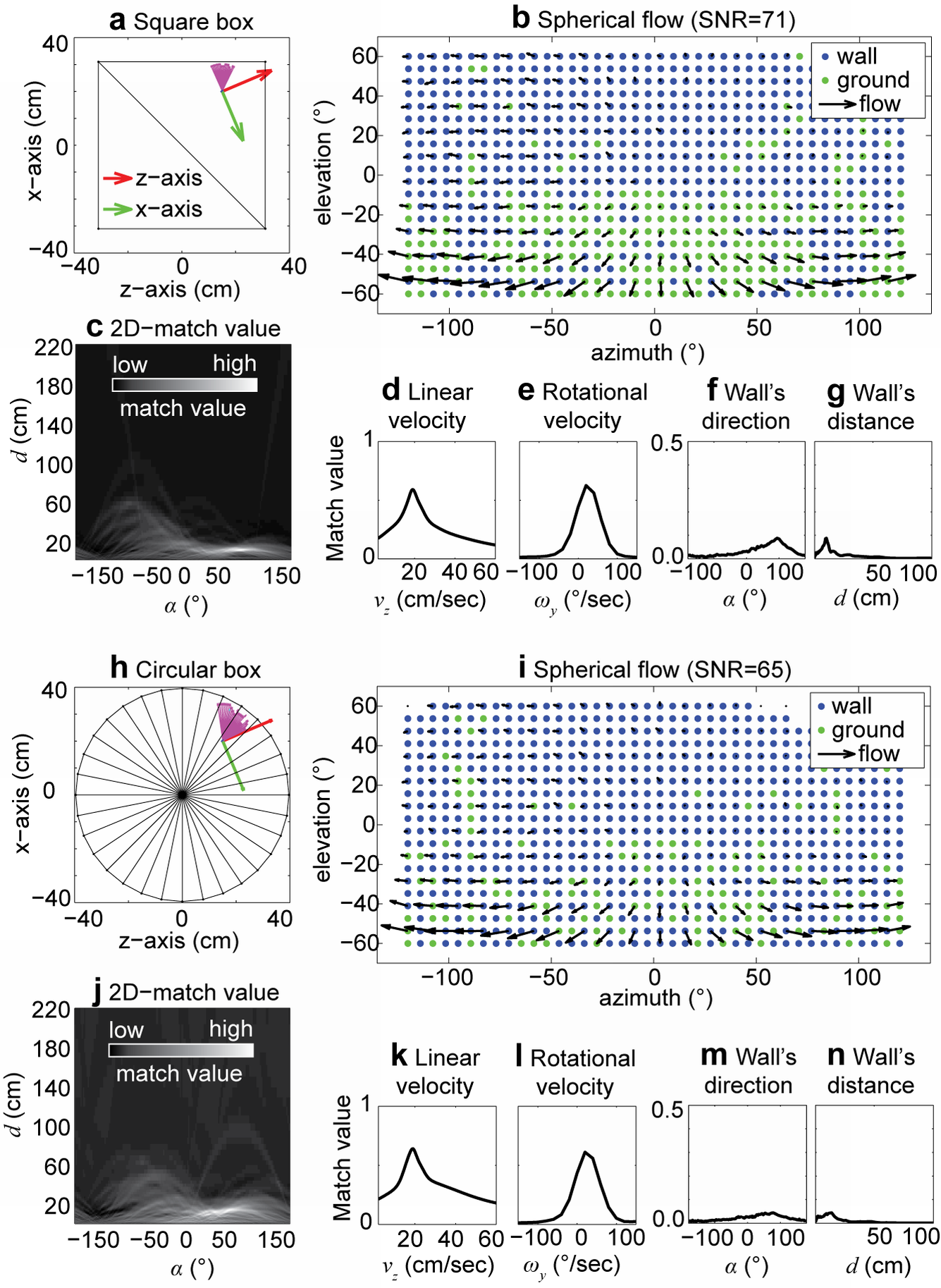

Supplement: Figure S1 — Examples of wall-ground segmentations, distance, and direction estimation of walls for analytical flow superimposed with additive, independent Gaussian noise. Flow with noise is defined as with Nθ and Nϕ drawn from a normal distribution with zero mean and σn as standard deviation. This type of noise strongly influences the quality of the segmentation and, thus, influences the other estimations based on this segmentation. In both examples the rat is positioned at x0 = 20 cm, y0 = 3.5 cm, z0 = 15 cm, has the head direction ϕ = 23°, and the self-motion vz = 19 cm/sec and ωy = 25°/sec. a) Square box with the coordinate system. The distance estimates are indicted by magenta arrows. b) Spherical flow field with noise (σn = 5°/sec) and wall-ground segmentation. c) 2D match values as calculated from samples that are indicated as originating from a wall, these are the blue dots in b). Low match values are encoded by low intensity and high match values by a high intensity, see also the inset with the color code. d–g) 1D match values for the linear and rotational self-motion and the direction and distance of walls. The latter two curves are computed as the maximum response from the 2D match value function of c), whereas the maximum is computed for the dimension not shown. The estimated self-motion is vz,est = 19 cm/sec and ωy,est = 24°/sec and the mean distance error is 1.15 cm with a standard deviation of 0.52 cm. h) Circular box with coordinate system and distance estimates of the wall depicted by the magenta arrows. i) Spherical flow with noise (σn = 15°/sec) and wall-ground segmentation for this circular box. j) 2D match values. k–n) Match value functions for velocities of the camera and direction and distance of walls. The estimated self-motion is vz,est = 19 cm/sec and ωy,est = 25°/sec and the mean distance error is 3.03 cm with a standard deviation of 1.29 cm. The distance error in the circular cage is higher due to the assumption about a planar approximation for each s [file pcbi.1002553.s001.tif]

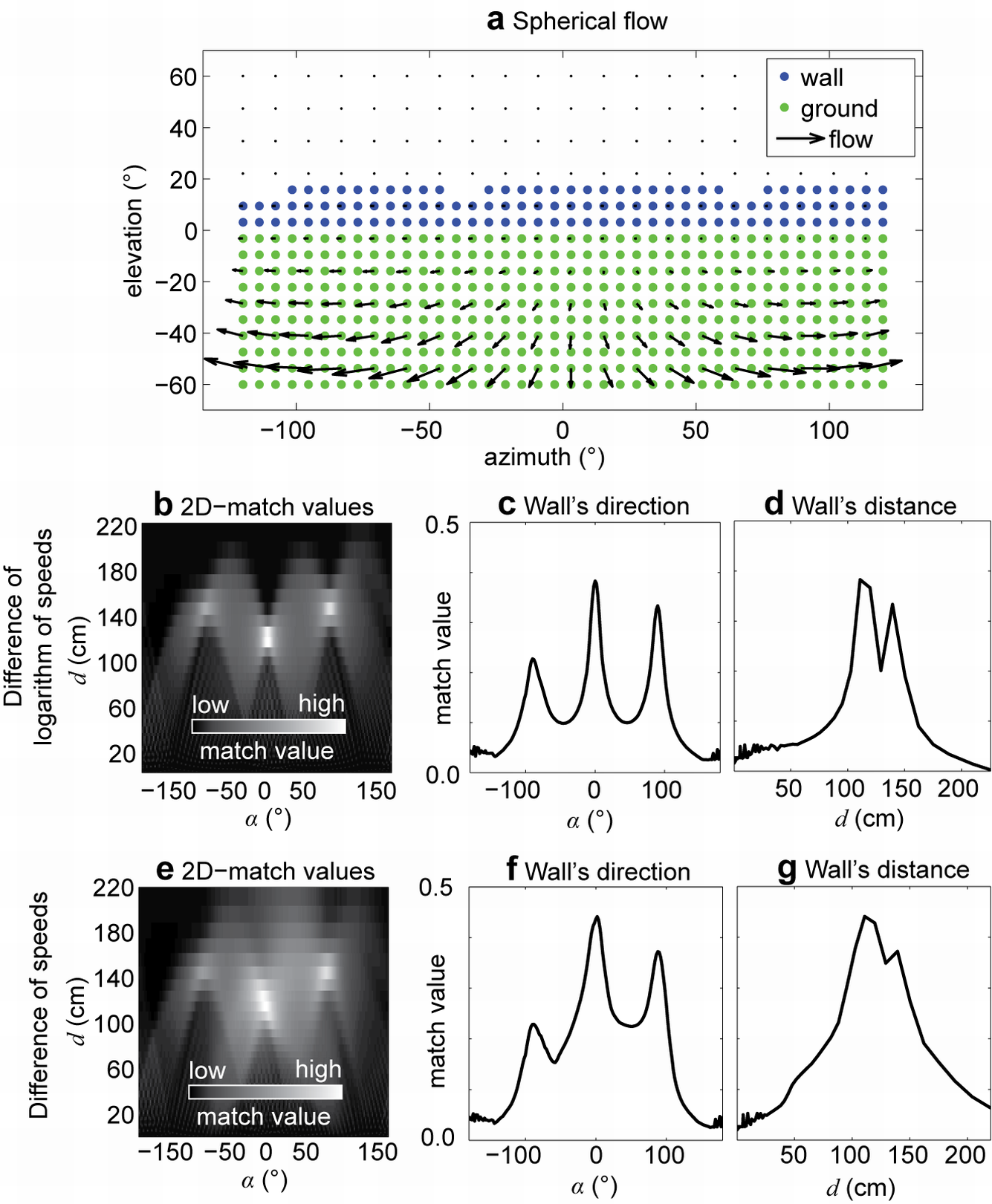

Supplement: Figure S2 — Taking the logarithm of the speeds of input and template flow vectors as the argument for a Gaussian function provides a “sharper” tuning compared to taking only the difference of speeds without the logarithm. a) Shows the spherical flow field and the detected wall-ground segmentation. b) 2D match values for the matching using the log-speed difference shows three clearly distinctive high intensity regions. These are better visible in the break-down into 1D curves c) for the wall's direction taking the maximum of all distances and d) the wall's distance taking the maximum of all directions. The mean error in distance estimates is 2.76 cm with a standard deviation of 1.81 cm. e) 2D match values for the matching using the speed difference without applying the logarithm. Compared to b) the matching occurs fuzzy, also visible in the break-down in the matching for f) the walls' directions and g) the walls' distances. In this case the mean distance error is 3.74 cm with a standard deviation of 3.85 cm and, thus, higher than in b). This example uses a rectangular box 250 cm×280 cm with 50 cm high walls and the rat's position is x0 = 0 cm, y0 = 3.5 cm, z0 = 10 cm with the head direction ϕ = 15°. (TIF) [file pcbi.1002553.s002.tif]

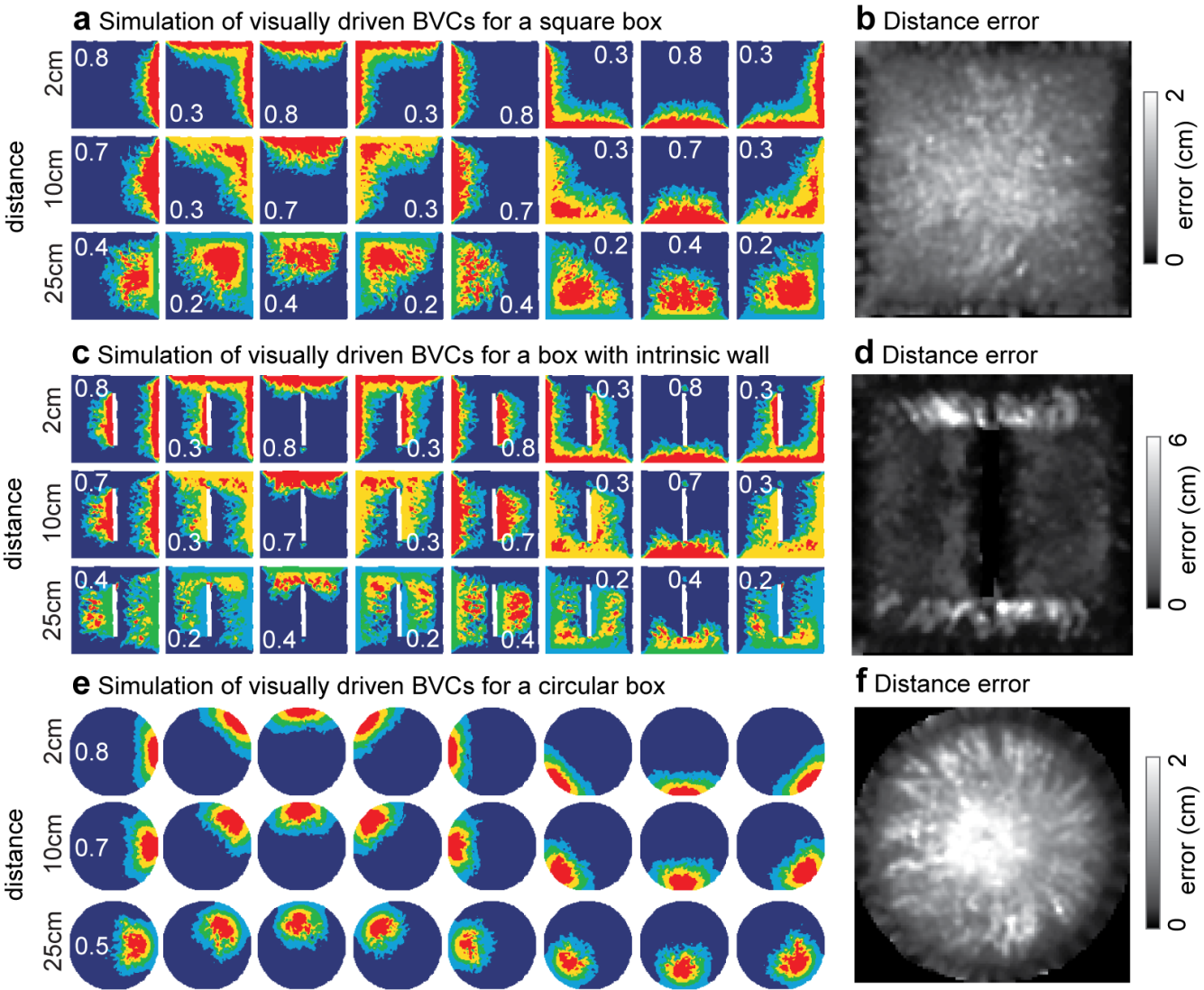

Supplement: Figure S3 — A tilt angle of 30°, that is the angle between the optical axes compared to the horizontal parallel to the ground, does no change firing fields qualitatively; however, the error at larger distances is larger compared to a zero tilt angle. a) Simulated BVC firing for a square box with distance and direction estimates for walls provided from our template model. b) Mean distance error in the two-centimeter range. c) Simulated BVC firing for the same square box as in a) but with an additional intrinsic wall. Firing appears at any wall of a specific distance and allocentric direction the cell is tuned for. d) Mean distance error in the range of zero to six centimeters. e) Simulated BVC firing for a circular box. f) Mean distance error in the two-centimeter range. (TIF) [file pcbi.1002553.s003.tif]
